# Lack of effects of a single session of cerebellar transcranial direct current stimulation (tDCS) in a dynamic balance task

**DOI:** 10.1007/s00415-020-09712-8

**Published:** 2020-01-31

**Authors:** K. M. Steiner, W. Thier, G. Batsikadze, N. Ludolph, W. Ilg, D. Timmann

**Affiliations:** 1 Department of Neurology, Essen University Hospital, University of Duisburg-Essen, Hufelandstr. 55, 45147 Essen, Germany; 2 Cognitive Neurology, Section Computational Sensomotorics, Hertie Institute for Clinical Brain Research and Center for Integrative Neuroscience, Eberhard Karls University, Tübingen, Germany

Dear Sirs,

There is ongoing interest in using transcranial direct current stimulation (tDCS) for clinical applications. Easy application, few side-effects and low costs contribute to its widespread use. For many years tDCS studies focused on M1 stimulation and its application in stroke patients [1, 2]. Only recently the cerebellum became of increasing interest as a stimulation target [3]. Some studies suggest favorable effects in patients with cerebellar ataxias [4, 5]. Initial expectations, however, have been muted because tDCS effects do not appear robust and frequently lack reproducibility. A publication bias towards positive results may have contributed to exaggerated expectations in the field [6]. We therefore believe that it is of interest to report negative findings.

**Fig. 1:**
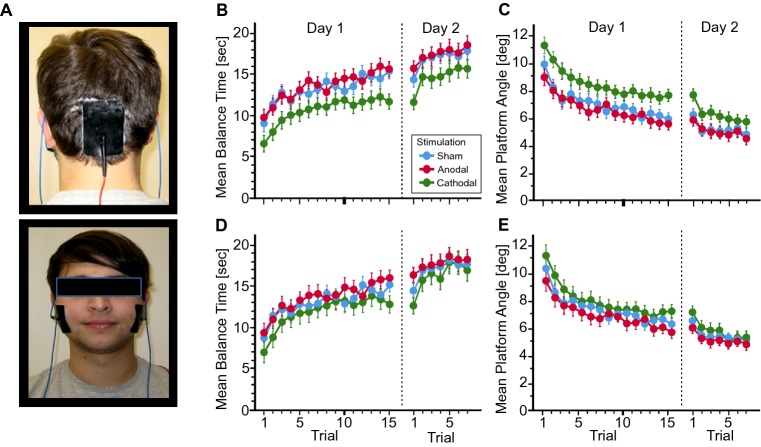
**a** Electrode montage. **b**, **d** Mean balance time and standard error and **c**, **e** mean platform angle and standard error **d**, **e** across trials in the three stimulation groups (sham: blue, anodal: red, cathodal: green). **b**, **c** All participants. **d**, **e** Participants taller than 185 cm were excluded. In the cathodal group five participants were taller than 185 cm, in the anodal group three participants and in the sham group one. Note that position of feet was fixed, and the task was more difficult for taller participants

We tested 48 young and healthy participants (24 male, 24 female, aged 20–29 years, mean age 23.6 years). Participants were pseudorandomly assigned to one of three stimulation groups (anodal, cathodal or sham stimulation) based on a pre-prepared allocation list. Whole body balance training was performed on a Lafayette Instrument 16030 Stability Platform^®^ (for more details see [7]). On the first day of training, participants performed 15 trials [8] and on the second day seven trials. Each trial lasted 30 s. Cerebellar tDCS was applied on the first day during training. Current intensity was set at 2.8 mA [9]. The cerebellar electrode (7 cm height × 5 cm width) was centered at the inion in a vertical orientation (upper edge 2.5 cm above the inion; Fig. 1a). Two return electrodes (5 cm × 5 cm) were placed over the buccinators muscles [7]. Mean platform angle and mean balance time (defined as the total time for each trial with the platform held between − 5° and 5°) were assessed [7].

All participants showed significant learning effects across the 22 trials (indicated by a significant decrease in the mean platform angle and significant increase in the mean balance time; trial effects, *p* values < 0.001; *η*^2^ = 0.57/0.49 (mean platform angle/mean balance time); ANOVA with repeated measures; Fig. 1b, c). The cathodal group performed below the sham and anodal groups [group effect—mean platform angle: *F*(2,45) = 3.98, *p* = 0.026; *η*^2^ = 0.15; mean balance time: *F*(2,45) = 3.12, *p* = 0.054; *η*^2^ = 0.12] with no significant difference between learning rates between groups [mean platform angle/balance trial × group interactions; *F*(19.5, 439.5) = 0.97, *p* = 0.49; *η*^2^ = 0.04/*F*(21.1, 474.6) = 0.64, *p* = 0.89; *η*^2^ = 0.03]. Because body height had a significant worsening effect on task performance (mean platform angle: *R* = 0.4, *p* = 0.004; mean balance time: *R* = − 0.38, *p* = 0.008), analysis was repeated with participants taller than 185 cm being excluded (Fig. 1d, e). The significant group difference did not remain [mean platform angle: *F*(2,36) = 0.9, *p* = 0.42; *η*^2^ = 0.05; mean balance time: *F*(2,36) = 0.64, *p* = 0.53; *η*^2^ = 0.03].

The present findings are in line with two previous studies of our group showing no cerebellar tDCS effects in the same complex whole body dynamic balance task in both young [7] and elderly participants [10]. In the present study the orientation of the cerebellar tDCS electrode was changed. A vertical orientation was used instead of a horizontal orientation. The rationale of this change was to accentuate stimulation of cerebellar midline structures known to be involved in posture and balance functions. Again, no significant tDCS effects on learning the balance task were observed. Studies investigating the same dynamic balance task applying anodal tDCS over the primary motor cortex reported also negative findings, whereas application over the supplementary motor area impeded learning [8, 11]. Negative findings and lack of reproducibility of cerebellar tDCS effects have also been reported in reach adaptation [12–14] and eyeblink conditioning [15], two other motor learning tasks known to be cerebellar dependent. Cerebellar tDCS effects on motor learning appear to be limited, at least based on a single session (see [13, 14] for discussion of possible reasons). This lowers expectations that cerebellar tDCS will be able to enhance the effects of physical therapy in patients with cerebellar ataxias.
